# Mycoprotein as chicken meat substitute in nugget formulation: Physicochemical and sensorial characterization

**DOI:** 10.1002/fsn3.3354

**Published:** 2023-04-17

**Authors:** Fataneh Hashempour‐Baltork, Behrooz Jannat, Manouchehr Dadgarnejad, Adel Mirza Alizadeh, Kianoush Khosravi‐Darani, Hedayat Hosseini

**Affiliations:** ^1^ Halal Research Center of IRI, Iran Food and Drug Administration Ministry of Health and Medical Education Tehran Iran; ^2^ Department of Food Safety and Hygiene, School of Public Health Zanjan University of Medical Sciences Zanjan Iran; ^3^ Department of Food Science and Technology, Faculty of Nutrition Science and Food Technology, National Nutrition and Food Technology Research Institute Shahid Beheshti University of Medical Sciences Tehran Iran

**Keywords:** chicken nugget, eco‐friendly, meat alternative, mycoprotein, optimization, vegan

## Abstract

The aim of this study was to replace chicken breast by mycoprotein in nuggets and optimizing the sensory and technological properties. In the first step of the study, 14 formulations were prepared by mixture design to evaluate the impact of three binding agents (as independent variables): soy protein isolate, phosphate, and carrageenan on sensory properties. Then, the optimized formulation of mycoprotein nugget (with higher acceptability) was characterized and compared to chicken nugget (control) from texture, color, and physicochemical aspects. The texture attributes including hardness, springiness, cohesiveness, and chewiness of the optimized sample (1.37 kg, 0.70 mm, 0.56, and 0.53 kg.mm) had no significant difference (*p* > .05) compared to control. Based on the results, optimized sample had a lower lightness and yellowness (a*, b*, and L* were 3.06, 18.62, and 59.23, respectively) rather than the similar value of the control (2.20, 21.27, and 79.10, respectively), which indicated carrageenan did not lead to any significant impact (*p* > .05) on the color. Also, mycoprotein nugget showed 33% lower cooking loss in comparison to control. Moisture, protein, lipid, and ash in optimized sample were 57.9 ± 1.9, 24.1 ± 1.0, 13.2 ± 1.2, and 2.1 ± 0.5, respectively. Investigation on physicochemical properties shows an acceptable characterization in optimized sample in comparison to control. The results of this study present an opportunity to produce nonmeat nuggets with similar texture and acceptable sensory and technological characteristics by using mycoprotein as meat alternative. The production of mycoprotein is eco‐friendly, not dependent on climate (flood and drought) and landscape limitation, which is an important aspect in meat alternatives in the near future.

## Graphical abstract

In the field of single cell protein production, various research have been reported, however, few works have been focused on Replacement of meat in nugget. Some evaluations about nutritional and sensory aspects on the final product was conducted. Anyway, as you know as a frontier researcher in many fields of food science, vegetarian are fast growing and also biotechnology based foods are the next decay human hope and one of critical aspects of biotechnology would be in the section of Food technology.

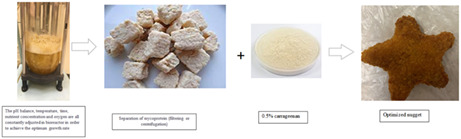

## 1 INTRODUCTION

The consumption of ready‐to‐eat and prepared precooked meals is increasing due to the lack of time for cooking food. However, the consumption of these foods has led to health concerns in different aspects (Bakhtiary et al., 2016; Mahros et al., 2021). Awareness on increasing health risks associated with inappropriate diets led to increasing demand for healthy food without sacrificing taste or texture. On other hand the meat production industry has been trying to find suitable ingredients such as fibers, bioactive compounds, and minerals to produce the healthier products. The use of meat alternatives, which contains protein and dietary fiber in meat products without change in texture and acceptability besides cost reduction, is the priority of food industry (Andreani et al., 2023). Besides health concerns, high meat consumption can affect climate change as well (Tilman & Clark, 2014).

Growing global population, concerns regarding the food crisis or accessibility of healthy foods, and also environmental issues have resulted in advanced studies on the production of single‐cell protein such as mycoprotein as a meat substitute (Zeng et al., 2023). Mycoprotein includes protein, fat, fiber, and carbohydrate (45%, 13%, 25%, and 10% w/w dry weight, respectively), as well as dietary fibers and micronutrients (e.g., Zn, Se, Fe, and also vitamin B_12_; Finnigan et al., 2017, Hashempour‐Baltork, Khosravi‐Darani, et al., 2020; Nyyssölä et al., 2022); its biological value was comparable with casein and protein digestibility score is around 1.0. Mycoprotein has sufficient content of essential amino acids and suitable ratio of unsaturated:saturated fatty acids (2:1). Furthermore, all aspects of safety and nutritional properties of mycoprotein have been studied (Hashempour‐Baltork, Khosravi‐Darani, et al., 2020). It is “Generally Recognized as Safe” (Hashempour‐Baltork, Khosravi‐Darani, et al., 2020; US FDA, 2002), and there is no contradictory report on the negative impact of mycoprotein consumption on growth (Bratosin & Vodnar, 2021) or health concern following short‐ and/or long‐term consumption (Finnigan et al., 2017). Also, due to the consumption of wastes, such as carbon, and energy source of microorganisms, single‐cell protein production could be considered as an eco‐friendly strategy (Salazar‐López et al., 2022).

A main concern in the reformulation of meat products is maintaining sensory characteristics like firmness, rubberiness, juiciness, and color similar to control. Generally, reformulation of meat products is conducted by using binding water substances to improve juiciness and reduce dryness and rubberiness. For improving the formulation of products, a mixture design methodology could be applied as a practical tool (Afshari et al., 2015). There are various aiding process materials for the reformulation of foods. For instance, soy proteins can set a heating gel and trap moisture. The gel formation leads to improvement in the juiciness and firmness of the product and also lessens the cooking loss throughout frying (Kyriakopoulou et al., 2021). Phosphates improve the gelling and emulsifying properties which result in developing the overall quality of meat products (Goemaere et al., 2021). Carrageenan is used as a processing aid substance in processed meat production for improving yield, texture, and appearance (Amini Sarteshnizi et al., 2015). Addition of <2% and 0.4% of κ‐carrageenan and polyphosphate, respectively, significantly affected product cooking yield and textural properties (Hsu & Chung, 2001). To the best of our knowledge, there is no report in the literature about incorporation of mycoprotein as substitution for meat in chicken nugget formulation.

Hence, this study has been designed to use mycoprotein as a meat substitute in chicken nuggets with the aim of producing a healthy and eco‐friendly meal suitable for vegans. To determine the optimum mixture combination, a response surface mixture design (D‐optimal) was used for three binding agents (as independent variables): soy protein isolate, phosphate, and carrageenan. Then, the optimized formulation was characterized from texture, color, and physicochemical properties.

## 2 MATERIALS AND METHODS

### 2.1 Nugget formulation and processing

For the nuggets processing, the chicken breast was purchased from a local market in Tehran, minced in a grinder (model WWB 200; Laska), and mixed to homogeneity with other components. The nugget mass formulation was prepared as given in Table 1 (as a control treatment).

**TABLE 1:** Nugget ingredients.

	Content (% w/w)
Main ingredient	
Chicken breast/mycoprotein	70
Onion	14
Texturized soy protein	6.3
Frying oil	3.4
Salt	1.3
Black pepper	1
Wheat flour	4
Batter ingredient	
Breading	91
Sauce (including pasteurized egg, salt, and pepper)	9

The mycoprotein mass produced from *Fusarium venenatum* was according to the authors previous study (Hashempour‐Baltork, Hosseini, et al., 2020). In the first step, the *F. venenatum* was grown by using a stirred tank reactor fermentation system and Vogel media. Next, the biomass of the fungus was heat treated (65°C, 15 min) to reduce ribonucleic acid content to permitted levels. In the final step, the suspended mycelium was recovered by centrifugation and the supernatant was used as mycoprotein (Hashempour‐Baltork, Hosseini, et al., 2020). Portions (around 25 g) of the mass were formed into star nuggets and then glazed with the batter coating and breading (Table 1). After frying at 180°C, the samples were packed in polyethylene bags and kept at −18°C. All the analyses were carried out after frying (ready for eating).

### 2.2 Experimental design

A mixture trial is a “special type of response surface methodology” in which the independent factors are the components of a mixture (here ingredients) and the dependent variables are a function of the individual component. The aim of the experimental design is to find a suitable combination of components to achieve responses with desired values. In this study, to maximize the acceptability by a sensory evaluation panel of mycoprotein nugget, design expert D‐optimal was preferred because it permitted several levels of the components to be examined with just a fraction of runs contrary to factorial design methods. The independent variables were soy protein isolate, kappa‐carrageenan, and phosphate. The total amount of independent variables reduced from the mycoprotein content in the formulation. The content range of independent variables chosen was based on legislation and literature. The dependent variable (response) was the objective measurement of overall acceptability (Table 2).

**TABLE 2:** Experimental design of the three components in mycoprotein nugget formulation.

Standard order	Independent			Response
	Soy protein isolate (%)	Carrageenan (%)	Phosphate (%)	Sensory evaluation score
1	0	0	0.5	4
2	0	0.25	0.25	4
3	0	0.5	0	5
4	5	0.5	0	4.9
5	5	0	0.5	3.3
6	5	0.25	0.25	4
7	0	0.375	0.125	4.7
8	5	0.375	0.125	4.7
9	0	0.125	0.375	3
10	5	0.125	0.375	3.4
11	0	0	0.5	3.2
12	0	0.5	0	5.1
13	5	0.5	0	4.85
14	5	0	0.5	3.2

### 2.3 Sensory evaluation

The sensory panel consisted of 11 trained consumers (males and females between 30 and 40 years) from the “National Nutrition and Food Technology Research Institute of Iran.” The treatments were assessed in fried samples to find out whether the consumers “liked or disliked” the experimental design treatments. The acceptance test of the nuggets was conducted on a “7‐point hedonic scale” ranging from 1 (disliked much) to 7 (liked much) for the attributes flavor, odor, taste, and overall acceptability. The samples were cut into 2 cm^3^ portions and each of them was covered with the foil and labeled with a random three‐digit code. The nuggets were held warm until the evaluation time. Nuggets were offered to consumers in a random order to avoid any bias, and water and biscuit were used as a palate cleanser (Carvalho Barros et al., 2020).

### 2.4 Textural properties

The texture profile analysis of fried nugget was done by Stable Microsystems Texture Analyzer (Model TA.XT plus) with a test cell (50 mm × 50 mm) working with a 2‐mm/s crosshead speed and 50% of strain. The distance, return speed, and contact force were 50 mm, 2 mm/s, and 20 g, respectively. Before the assessment, the coating batter of the sample was detached to avoid probable interference. The attributes such as hardness (kg), springiness (mm), cohesiveness, and chewiness (kg.mm) were calculated (Kamani et al., 2019).

### 2.5 Color

A colorimeter (Hunter Lab, Minolta CR‐400) was used for measuring the color parameters. Samples were used in replicates and evaluation was conducted at 25°C by scanning the L*, a*, and b* parameters, which represent the lightness, the red‐green, and the blue‐yellow spectrum, respectively. Before the measurement, the samples refrigerated at 4°C were thawed and the readings of the mass were taken by being cut in half.

### 2.6 Proximate composition analyses of optimized nugget

The proximate composition of nuggets including moisture, protein, lipids, and ash (of the three samples) was determined by official methods (AOCS, 2017). Moreover, the cooking loss was determined based on the method by Carvalho Barros et al. (2020) with minor modifications.

### 2.7 Statistical analysis

A fully randomized design was applied in this experiment. One‐way analysis of variance (ANOVA) was used to determine significant differences between burger formulation groups by the IBM SPSS version 21.0. The data were analyzed using generalized linear model (GLM) procedure, the least squares differences (LSD) were utilized for comparison of mean values among formulations, and the Duncan's multiple range test was performed to identify significant differences between the treatments. The sensory test was evaluated with an ANOVA to study the effect of mycoprotein added and when the differences were significant (*p* < .05). A Tukey's test at a significance level of *p* < .05 verified the differences between the pair of groups. Descriptive data were reported as mean ± standard deviation for the three replicates.

For formulation of new product, a mixture design was performed and studied by Design Expert (v. 7.6.1, Stat‐Ease Inc.). The effect of ingredient combination on nugget features was analyzed by a D‐optimal mixture method (Table 2). The constraints (low/max) for each component were set previously in initial trials. Within this study, 14 treatment combinations with three independent variables were employed. The mixture design was generated to enable the investigation of the interaction effects of ingredients on the acceptability of mycoprotein nugget.

## 3 RESULTS AND DISCUSSION

### 3.1 First phase (experimental design)

Table 2 demonstrated the experimental design and sensory assessment scores as its response. Table 3 indicates the model characteristics, significance, lack of fit values, coefficient R (Andreani et al., 2023), interaction, and linear effects (Table 3). According to Equation (1), obtained by software, both carrageenan and phosphate have a positive effect on the overall acceptability of formulated nuggets, but carrageenan has the highest effect (5.04).

(1) $$Y=5.04\;\text{carrageenan}+3.34\;\text{phosphate}-1.03\;\left(\text{carrageenan}\times \text{phosphate}\right)$$

**TABLE 3:** Analysis of variance of the regression models and regression coefficients.

Parameter	Coefficient	*F*	*p*	*R* ^2^	*R* ^2^ adjust
Constant	–	34.66	<.0001	.86	.86
Linear mixture		67.63	<.0001		
Carrageenan–phosphate		1.69	.2206		
Lack of fit	–	1.25	.4372		

Phosphates develop the functional role of proteins in different ways such as dissociation of the actomyosin complex, increase in pH of products which leads to increasing the gap between final pH and isoelectric point, rising the electrostatic repulsive forces which caused more water trap in these gaps, and rise the ionic strength which leads to protein activation. In contrast, carrageenan is arranged in the interstitial spaces of the protein network, upon cooling, and gel fragments are formed which hold water inside them (Petracci et al., 2013). According to Table 2, the substitution of chicken meat and mycoprotein by using 0.5% carrageenan lead to higher acceptability. Thus, it is possible to develop new, nutritious, and healthy food for vegans as well as in drought period at lower cost.

### 3.2 Second phase (characterization)

According to the results of the experimental design, number 12 of Table 2 (mycoprotein nugget + 0.5% carrageenan) was chosen as the best treatment (optimized nugget). Characterization and comparison tests were carried out with three samples of chicken nugget (control), mycoprotein nugget, and optimized nugget (mycoprotein nugget + 0.5% carrageenan).

#### 3.2.1 Textural evaluation

The mechanical properties of cooked samples are reported in Table 4. Mycoprotein nugget has the lowest (*p* ≤ .05) content of hardness, springiness, cohesiveness, and chewiness. It was found that lower force was needed to chew a mycoprotein nugget compared to the chicken and optimized samples. This is possibly due to the weaker network of mycoproteins, which results in a decrease in the resistance of the product to compression. The mycoprotein nugget indicated the lower (*p* ≤ .05) values for springiness and cohesiveness. Similarly, it was found that springiness values are lower when soy protein isolate is used as a meat alternative in meat batter (Youssef & Barbut, 2011). Moreover, a lower level of cohesiveness, hardness, springiness, and gumminess was observed in the substitution of meat for plant proteins in chicken sausage (Kamani et al., 2019). They mentioned that nonmeat proteins can retain further water and fat contents, which can fill the interstitial spaces within the protein matrix and reduce the springiness (Kamani et al., 2019; Youssef & Barbut, 2011).

**TABLE 4:** Textural analysis of the three different nuggets.

Treatment	Hardness (kg)	Springiness (mm)	Cohesiveness	Chewiness (kg.mm)
Chicken nugget (control)	1.41 ± 0.14^a,^ *	0.73 ± 0.12^a^	0.58 ± 0.11^a^	0.59 ± 0.20^a^
Mycoprotein nugget	1.15 ± 0.08^b^	0.61 ± 0.09^b^	0.42 ± 0.14^b^	0.30 ± 0.17^c^
Optimized sample**	1.37 ± 0.12^a^	0.70 ± 0.19^a^	0.56 ± 0.12^a^	0.53 ± 0.21^a^

* Mean ± SD. Different letters represent significant differences (*p* < .05)

** Optimized sample: mycoprotein nugget + 0.5% carrageenan.

As previously pointed out, the organoleptic qualities of meat products are vital, especially in processed meat products where perceived texture, for example, bite, juiciness, and tenderness are of utmost importance to the consumer. Certainly, there was no difference (*p* > .05) between chicken and optimized samples in all textural attributes. The water binding functionality of carrageenan plays a key role here. A variety of textures can be achieved with carrageenan, thus a range of textures can be created as appropriate for the end product (Hotchkiss et al., 2016).

#### 3.2.2 Color

Colorimetric assessment data are available in Table 5. These results indicated that replacing mycoprotein leads to a reduction in lightness (L*), yellowness (b*), and an increase in redness (a*) parameters. Incorporation of carrageenan to formulation of product did not lead to any significant difference in color evaluation parameters. Types of spices and the level of hemoproteins may have a high effect on the color indices of the end product. In this study, black pepper was used in formulation, but to decrease the darkness of the product, white pepper can be a substitute. The color of the meat product is often noticed to be the most important point for consumers' intention to purchase. Uncooked meat products have a characteristic color, which is usually bluish‐white to yellow for poultry, bright cherry red for beef, and reddish pink for pork. The color of these products can change by cooking because the protein (mainly myoglobin) which is responsible for these characteristic colors of meat undergoes chemical changes during cooking (Bohrer, 2019). A similar concept can be used for meat analog products indicating the importance of the color and the color change during cooking. Similar color attributes should be seen before, during, and after cooking for analogous meat products and the meat products that they are simulating (Kyriakopoulou et al., 2019).

**TABLE 5:** Colorimetric evaluation of the three different samples.

Treatment	*a* *	*b* *	*L* *
Chicken nugget (control)	2.20 ± 0.02^b^ *	21.27 ± 0.03^c^	70.1 ± 0.04^a^
Mycoprotein nugget	3.02 ± 0.03^a^	18.57 ± 0.03^b^	59.18 ± 0.05^b^
Optimized sample^†^	3.06 ± 0.02^a^	18.62 ± 0.02^b^	59.23 ± 0.02^b^

* Mean ± SD. Different letters represent significant differences (*p* < .05).

^†^ Optimized sample: mycoprotein nugget + 0.5% carrageenan.

#### 3.2.3 Physicochemical evaluation

The proximate analyses of different nuggets were shown in Table 6. The main concern of the manufacturer of meat alternatives is to generate a product that has overall physicochemical and nutrient properties of the traditional meat product (Hashempour‐Baltork, Khosravi‐Darani, et al., 2020; Kyriakopoulou et al., 2019). From a functional and nutritional perspective, it is important to maintain similar content of lipids and moisture.

**TABLE 6:** Proximate analysis of the three different nuggets.

Treatment	Moisture (%)	Protein (%)	Lipid (%)	Ash (%)	Cooking loss (%w/w)
Chicken nugget (control)	54.5 ± 1.9^c,^ *	27.2 ± 0.19^b^	13.9 ± 1.3^c^	2.5 ± 0.15^a^	4.50 ± 0.22^a^
Mycoprotein nugget	56.1 ± 1.8^b^	24.5 ± 0.8^b^	13.00 ± 1.08^b^	2.02 ± 0.8^c^	3.70 ± 1.02^b^
Optimized sample**	57.9 ± 1.9^a,^ *	24.1 ± 1.0^b^	13.2 ± 1.2^b^	2.1 ± 0.5^b^	3.00 ± 1.05^c^

* Mean ± SD. Different letters represent significant differences (*p* < .05)

** Optimized sample: mycoprotein nugget + 0.5% carrageenan.

The moisture content has an increase (*p* ≤ .05) in the mycoprotein nuggets, while the optimized sample has the highest moisture content. Carrageenan chains involve the units of d‐galactose and 3,6‐anhydro‐glactose. These units are connected by α‐1,3 and β‐1,4 glycosidic links. Sulfate groups, which affect the final properties of the gel, are also involved in their structure. Sulfate group content decreases solubility temperature and also gel strength (Bartlová et al., 2021). Besides, carrageenan is incorporated into the brines that are used in the production of processed meats, either during the tumbling stage of reformed products or into the injection brines that are used for whole muscle products (particularly hams) (Hotchkiss et al., 2016).

The lipid and protein contents also decreased (*p* ≤ .05) in mycoprotein‐containing samples. Addition of carrageenan has no significant effect (*p* > .05) on the protein and lipid contents. Results indicated that the mycoprotein‐containing samples have lower ash rather than chicken nugget. The optimized nugget, in comparison to mycoprotein nugget, has higher ash content, which could be related to carrageenan content. However, there are significant (*p* ≤ .05) differences in moisture, protein, and lipid values of reformulated and control samples, but the values were acceptable according to frozen chicken product specifications (ISIRI, 2007). These findings are in accordance with the earlier studies that have considered mycoprotein as a future nutritious and healthy protein (Finnigan et al., 2019).

The cooking loss values were found to be reduced significantly (*p* ≤ .05) by using mycoprotein as a meat alternative. The highest and lowest (*p* ≤ .05) cooking loss is related to chicken nugget and optimized nugget, respectively. Cooking loss is defined as “the loss of water and soluble matter from meat during cooking” and indicates the ability of the system to bind water and fat after protein aggregation and denaturation (Aaslyng et al., 2003). Carrageenan improves gel strength and water binding which serves to improve important attributes such as cooking loss and purge, which not only affect the appearance of cooked and uncooked products but also have significant cost implications for producers (Hotchkiss et al., 2016). It indicates that the emulsions' network structure in nonmeat nuggets has a strong rule in maintaining the moisture during cooking. The cooking loss value in the control sample (chicken nugget) was significantly (*p* ≤ .05) more than other samples, which shows a weaker ability of meat proteins to bind the water in comparison to the mycoproteins. Several factors can affect the cooking loss such as the content and the type of fat in formulation, method of cooking, time, temperature, and additives used in emulsion and casing material (Choi et al., 2009).

## 4 CONCLUSION

This study was conducted to investigate the possibility of mycoprotein application as a meat alternative in the chicken nuggets. The results indicated that the mycoprotein substitution could improve the nutritional effects caused by higher valuable proteins (including essential amino acids) and lower lipids (mostly unsaturated fatty acids). Results indicated that the overall acceptability of optimized nugget (mycoprotein + 0.5% carrageenan) and chicken nugget were the same. Besides, the health, safety, price, and eco‐friendly factors of optimized nugget are better than chicken nugget.

There was no difference between the textural parameters of the optimized sample compared to the control. Carrageenan indicated no significant impact on the color of the product; however, sample containing mycoprotein had less lightness and yellowness rather than the control. It suggests that using white pepper in mycoprotein nugget can reduce the darkness of the end product. Moreover, the optimized sample indicated acceptable values for physicochemical properties which were close to the control sample. The cooking loss parameter was reduced by using the mycoprotein in samples, which indicates its economic advantages. Although the optimized sample containing carrageenan had the lowest cooking loss. Carrageenan is used in the food industry not only for its gel‐forming properties, but also for its stabilizing, emulsifying, and thickening properties.

This study was the first article considering the substitution of chicken with mycoprotein in chicken nugget and also the optimization of textural characteristics in mycoprotein nuggets, which is the main problem in the production of the meat‐free product.

## AUTHOR CONTRIBUTIONS

Fataneh Hashempour‐Baltork collected the data, wrote the draft, and conducted the work. Behrooz Jannat and Manouchehr Dadgarnejad contributed to the software, tool analysis, and revision of the draft. Adel Mirza Alizadeh contributed and analyzed the data. Kianoush Khosravi‐Darani and Hedayat Hosseini supervised the study, revised the draft, and contributed to the software and idea of research.

## CONFLICT OF INTEREST STATEMENT

The authors declare that they have no known competing financial interests or personal relationships that could have appeared to influence the work reported in this article.

## ETHICS STATEMENT

Not applicable.

## Data Availability

Data sharing not applicable to this article as no datasets were generated or analysed during the current study.
